# Circadian rhythms enable efficient resource selection in a human‐modified landscape

**DOI:** 10.1002/ece3.5283

**Published:** 2019-06-21

**Authors:** Manuela Fischer, Julian Di Stefano, Pierre Gras, Stephanie Kramer‐Schadt, Duncan R. Sutherland, Graeme Coulson, Milena Stillfried

**Affiliations:** ^1^ School of Ecosystem and Forest Sciences University of Melbourne Creswick Victoria Australia; ^2^ Conservation Department Phillip Island Nature Parks Cowes Victoria Australia; ^3^ Australian Wildlife Conservancy Parramatta Park Queensland Australia; ^4^ Department of Ecological Dynamics Leibniz Institute for Zoo and Wildlife Research Berlin Germany; ^5^ Berlin‐Brandenburg Institute of Advanced Biodiversity Research Berlin Germany; ^6^ Department of Ecology Technische Universität Berlin Berlin Germany; ^7^ Department of BioSciences University of Melbourne Parkville Victoria Australia

**Keywords:** correlated random walk, GPS‐telemetry, habitat selection, human disturbance, macropod, risks and benefits

## Abstract

Animals access resources such as food and shelter, and acquiring these resources has varying risks and benefits, depending on the suitability of the landscape. Some animals change their patterns of resource selection in space and time to optimize the trade‐off between risks and benefits. We examine the circadian variation in resource selection of swamp wallabies (*Wallabia bicolor*) within a human‐modified landscape, an environment of varying suitability. We used GPS data from 48 swamp wallabies to compare the use of landscape features such as woodland and scrub, housing estates, farmland, coastal areas, wetlands, waterbodies, and roads to their availability using generalized linear mixed models. We investigated which features were selected by wallabies and determined whether the distance to different landscape features changed, depending on the time of the day. During the day, wallabies were more likely to be found within or near natural landscape features such as woodlands and scrub, wetlands, and coastal vegetation, while avoiding landscape features that may be perceived as more risky (roads, housing, waterbodies, and farmland), but those features were selected more at night. Finally, we mapped our results to predict habitat suitability for swamp wallabies in human‐modified landscapes. We showed that wallabies living in a human‐modified landscape selected different landscape features during day or night. Changing circadian patterns of resource selection might enhance the persistence of species in landscapes where resources are fragmented and disturbed.

## 1 INTRODUCTION

Human land‐use change has led to habitat loss and fragmentation (Fahrig, 2003). Both scenarios result in an increase in spatial heterogeneity of resources, which are important for animals to survive (White, 1983). When animals select habitats, they have to consider factors such as access to food, water, mates and shelter and predator avoidance (Manly, McDonald, Thomas, McDonald, & Erickson, 2002) and habitat selection may improve the use and accessibility to these resources. However, habitat utilization depends on its availability (Manly et al., 2002) and the selection of habitat by an animal may involve a trade‐off between risks and benefits, such as increased food quality or quantity and predator exposure (Dupke et al., 2017; Lima & Dill, 1990), which may vary in time.

Human‐caused landscape fragmentation can provide novel and easily accessible resources for wildlife, such as high‐quality food and water, through an increase in agricultural activity and urbanization (Contesse, Hegglin, Gloor, Bontadina, & Deplazes, 2004). Nevertheless, land‐use change may cause human‐wildlife encounters to increase, such as harassment or predation of wildlife by domestic dogs (Banks & Bryant, 2007; Hughes & Macdonald, 2013), animal‐vehicle collisions (Haikonen & Summala, 2001; Olson et al., 2014), hunting (Madsen & Fox, 1995), and outdoor recreational activities (Taylor & Knight, 2003). Hence, animals selecting resources in human‐modified landscapes may encounter habitats that confer opposing benefits such as high‐quality food versus shelter. Understanding how animals select resources and persist in human‐modified landscapes is important for successful species management and conservation (Graham, 2001; Klar et al., 2008).

Resource acquisition is linked to an animal's daily foraging and resting rhythm (Lima & Dill, 1990; Rettie & Messier, 2000). For example, many herbivores forage in open grasslands and seek sheltered resting sites in adjacent vegetation (Bjørneraas et al., 2011; Dupke et al., 2017; Johnson, 1980). The European roe deer (*Capreolus capreolus*) accesses high‐quality food in grassland, but this exposes them to stress factors such as heat, increased predation risk, and human disturbance (Bonnot et al., 2012). To optimize this trade‐off of animals encountering landscapes with opposing benefits, it has been shown that herbivores alter their behavior through time (Lykkja et al., 2009; Markovchick‐Nicholls et al., 2008; Munns, 2006; Rettie & Messier, 2000). For example, moose (*Alces alces*) and red deer (*Cervus elaphus*) select habitats with high vegetation cover during the day to rest and frequently used pastures with less cover but better food quality during the night when they are less visible to predators and humans (Bjørneraas et al., 2011; Godvik et al., 2009). Further, it was shown that hunting pressure and the distance to roads and dwellings influenced habitat use of roe deer as they selected more sheltered habitats during daytime, when risks such as human disturbance are higher, and increased the use of open fields at night (Bonnot et al., 2012).

Differences in resource selection in human‐modified landscapes have also been found between sexes. Female grizzly bears (*Ursus arctos*) were found near roads more frequently than expected compared to males, suggesting that female bear‐vehicle encounters are higher (Graham, Boulanger, Duval, & Stenhouse, 2010). Further, female common noctule bats (*Nyctalus noctule*) traversed the land close to wind turbines on long flight paths, whereas males used a straight route between roosts and foraging areas, lowering the risks of colliding with turbines (Roeleke, Blohm, Kramer‐Schadt, Yovel, & Voigt, 2016).

A herbivore known to persist in highly human‐modified and fragmented landscapes is the swamp wallaby (*Wallabia bicolor*) (Ben‐Ami & Ramp, 2013), although most ecological studies have been conducted in their natural or seminatural habitats (DiStefano, 2007). Swamp wallabies are medium‐sized marsupials whose population has been increasing across their range (Allen & Mitchell, 2016; Menkhorst et al., 2016). The species natural habitat consists of forests and woodlands where wallabies often select areas with high shrub cover (Edwards & Ealey, 1975; Lunney & O'Connell, 1988; Troy, Coulson, & Middleton, 1992). Preferred food items include shrubs and forbs, but grasses, ferns, and sedges are also consumed, and grasses can dominate the diet in some situations (Hollis, Robertshaw, & Harden, 1986; Osawa, 1990; Di Stefano & Newell, 2008). Exotic plants are occasionally eaten in areas of human settlement (Osawa, 1990; Watson, 1993). Swamp wallabies are known to follow circadian patterns to avoid risks. For instance, they move into grassland during the night to graze and return to cover before sunrise (Edwards & Ealey, 1975). When cover is close, they sometimes occur in high abundances on farmland (Johnson, 1975), possibly resulting in high pasture loss as shown for other wallaby species (Smith et al., 2012). Further, Di Stefano, York, Swan, Greenfield, and Coulson (2009) and Swan, Stefano, Greenfield, and Coulson (2008) found that within timber production forests of varying harvesting ages, selection of food and shelter resources changed within a 24‐hr period. These studies took place in the swamp wallabies’ natural or seminatural habitat where resources are abundant. However, they are also known to persist in areas with human settlement sometimes even inhabit areas where human disturbances are high (Ben‐Ami & Ramp, 2013). Little is understood about their selection of resources within these landscapes, how they respond to disrupted and depleted resources, where competition for food and shelter might be high and why they are able to persist and even thrive in landscapes where species such as the red‐necked wallaby (*Macropus rufogriseus*) are known to fail (Zusi, 2010). Hence, swamp wallabies are an excellent model species to investigate habitat use and resource selection in such landscapes to better understand how animals persist within and respond to a highly human‐modified environment.

In this study, we used custom‐made Global Positioning System (GPS) trackers (Fischer et al., 2018) to collect fine‐scale movement data from 48 swamp wallabies to investigate the selection of resources in a fragmented and human‐modified landscape during the day and night. We analyzed resource selection by comparing the wallabies’ use of natural and more human‐disturbed landscape features to their availability. We hypothesized that wallabies use circadian behavioral cycles to access food and shelter while avoiding landscape features that may pose higher risks. To test this, we determined resource availability by using correlated random walks (CRW) based on real wallaby trajectories and compared the wallabies’ use of specific landscape features to the availability of these features during the day and night. Because the wallabies’ natural habitat consists of high shrub cover (Lunney & O'Connell, 1988; Troy et al., 1992; Wood, 2002), we expected that natural landscape features with high cover (woodland and scrub, coastal vegetation, and wetlands) are more likely to be selected than landscape features that may expose wallabies to higher risks (agricultural farmland, housing estates, waterbodies, and roads) and that the selection differs between day and night (Edwards & Ealey, 1975; Troy et al., 1992). In particular, we expected that natural landscape features are selected during the day and risky features are more tolerated at night. Further, we know that although some landscape features are avoided (e.g., roads), landscape features in close proximity might be important (e.g., roadside vegetation, Osawa 1989). Hence, we predicted that wallabies will prefer to be further away from landscape features that may represent higher risks, but that some of these features might be selected more, especially at night when perceived risks may be lower. Further, we predict that males would select landscape features that may pose higher risks more than females, because we know that male macropods are more often involved in fatal vehicle collisions, suggesting that they expose themselves to greater risk (Coulson, 1997).

## 2 METHODS

### 2.1 Study site

The study took place on Phillip Island (38°29′S; 145°15′E), located in southeast Australia and approximately 10,000 ha in size. Topography is mostly flat with the maximum elevation 112 m above sea level (Gliddon, 1958). The island's native grass and bushland areas have been cleared for agriculture by early settlers in the mid‐1800s (Head, 2000). The current landscape is dissected by roads and vegetation strips which are often dominated by swamp paperbark (*Melaleuca ericifolia*). To date, the island consists of approximately 20% of natural or replanted bushland, 20% is urbanized, 10% is coastal areas, and the remaining land is converted to agricultural farmland (Phillip Island Nature Parks, 2012). The canopy cover within the remnant bushland is dominated by native or revegetated eucalyptus woodlands, and austral bracken (*Pteridium esculentum) and hop goodenia (Goodenia ovata) are found in the lower cover*. Coastal shrubs include coastal tea‐tree (*Leptospermum laevigatum*), and open coastal areas are dominated by tussock‐grass (*Poa poiformis*), bower spinach (*Tetragonia implexicoma*), and seaberry saltbush (*Rhagodia candolleana*) (Sutter & Downe, 2000). The island is also a tourist destination visited by more than 1.8 million people annually, and the human population can exceed 40,000 during the peak holiday season (Bass Coast Shire Council, 2017). Humans are assumed to cause the main disturbance for swamp wallabies on the island, because the abundance of predators is low due to the removal of the introduced European fox (*Vulpes vulpes*) (Kirkwood, Sutherland, Murphy, & Dann, 2014).

### 2.2 Animal capture, handling, and data collection

**Figure 1:**
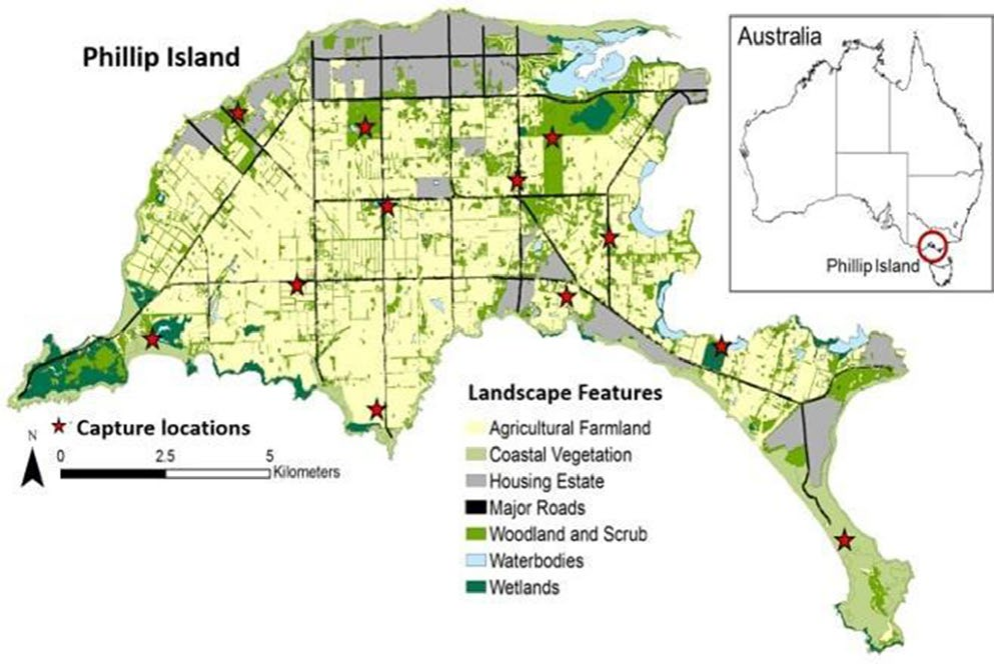
Study site Phillip Island, southeastern Australia, categorized in seven landscape features including wallaby capture locations

We captured 48 wallabies, 22 females, and 26 males, from January 2015 to March 2017 at 12 random locations within or near seven landscape types found on Phillip Island: agricultural farmland, coastal vegetation, housing estate, major roads, woodland and scrub, waterbodies, and wetlands (Figure 1). We captured wallabies using two methods. We trapped some in purpose‐built double‐layered traps (Di Stefano, Moyle, & Coulson, 2005) set in the late afternoon, baited with carrots, and checked the next morning. Trapped wallabies were sedated with an intramuscular injection of Zoletil 100 (0.5 mg/kg) (Virbac Australia, Sydney). We darted other wallabies on foot during dusk and dawn using a tranquilizer gun (Pneu‐Dart X‐caliber), using the same dose of Zoletil as reported above.

We fitted sedated adult wallabies with custom‐made GPS collars and ear‐tags. We scheduled the GPS collars with a 15‐min fix interval, 24 hr a day, seven days a week. GPS locations were sent remotely via the mobile phone network (Fischer et al., 2018). An overview of captured animals and tracking duration can be found Table 2 in Appendix 2. We discarded GPS locations which were collected while trapping was being conducted and within 8 hr after the sedation of an animal to ensure that the animal was fully conscious during data collection. In addition, we discarded fixes with horizontal dilution of precision >8, number of satellites <3, height > 100 m above sea level, and speed > 8 m/s using the online database Movebank (Wikelski & Kays, 2014). In total, we discarded 1,212 out of 24,160 locations, including two fixes which were classified as biologically implausible.

### 2.3 Habitat classification

To create a habitat map of Phillip Island, we used land‐use maps from Spatial Datamart (Department of Environment, Land, Water and Planning, 2016; downloaded 14 October 2014) including the Ecological Vegetation Classes such as wetlands and coastal areas. We added a tree cover index layer to identify areas ranging from low cover, representing open grassland, to high cover shown as woodland and scrub. The layer is based on shadow areas of vegetation, derived from color spectral analysis of aerial imagery. Further, we added a road and waterbody layer (Department of Environment, Land, Water and Planning 2016, downloaded 29 May 2013) and defined urban areas by using aerial images (Phillip Island Nature Parks, 2012). We classified the final map into seven landscape features: wetlands, woodland and scrub (natural or replanted bushland and roadside vegetation), coastal vegetation, housing estates, agricultural farmland, major roads, and waterbodies (Figure 1). We used the R statistical environment, version 3.4.1 (R Core Team, 2017) and ArcGIS, version 10.4.1 (ESRI, 2011) to create the map.

### 2.4 Data analysis

We followed a use versus availability design where we compared individual use of landscape features to available features within reach of the individual (Johnson, Nielsen, Merrill, McDonald, & Boyce, 2006; Manly, McDonald, Thomas, McDonald, & Erickson, 2007). We measured landscape feature availability using CRW (Bovet & Benhamou, 1988) which apply step lengths and turning angles of the original animal movement path to simulate random trajectories with sampling frequencies identical to that of the corresponding wallaby trajectory. To link the simulated movement track with the area used by each individual, we allowed CRWs within a buffer measuring one half of the square root of the 100% minimum convex polygon (Roeleke et al., 2016; Stillfried et al., 2017). We removed areas such as ocean and inland waterbodies from the buffer, as we assumed both to be unsuitable habitat for our study species. Within each individual's buffer, we simulated five CRWs with randomly selected starting points, using the R package “adehabitatHR” (Calenge, 2006). The total dataset consisted of the observed GPS locations (used; *n* = 22,947) and the simulated locations (available; *n* = 114,738) derived from the CRWs. We assigned each location to the underlying habitat map to extract its associated habitat feature (“sp package”; Pebesma and Bivand (2005)) and calculated the shortest distances to each of all seven landscape features (“rgeos package”; Bivand et al. (2017)). To identify differences in diurnal behavior, we specified day (dawn till dusk) and night (dusk till dawn) locations by applying sun ephemeris calculations using the function “crepuscule” in the R package “maptools” (Bivand & Lewin‐Koh, 2013).

#### 2.4.1 Landscape use model

To test whether wallabies select certain landscape features over others, we analyzed the data using a generalized linear mixed effects model (GLMM) with a binomial error distribution and logit link function in the R statistical environment (R Core Team, 2017) using package “lme4” (Bates, 2010). We used observed wallaby data (1) and CRW (0) as the binary response variable and accounted for unequal sample size between observed trajectories and CRWs by including a weights factor in our models. As predictor variables we chose the landscape features described above, except waterbodies as they were classified as unsuitable habitat for swamp wallabies. To evaluate circadian differences in landscape feature use, we included the categorical predictor variable “time” with the levels “day” and “night” in interaction with all six remaining landscape features in the model. We used the individual animal identifier (ID) as a random factor to account for interindividual variation. The same ID as for the observed trajectories was assigned to the five random walks. We used the model with the most parsimonious random effect structure to build model sets (Zuur, Ieno, Walker, Saveliev, & Smith, 2009) (Table 3 in Appendix 2) and used Akaike information criterion (AIC) controlled for small sample size (AIC_c_) and Akaike weights to indicate the degree of support within the model set (Bartón, 2013; Burnham & Anderson, 2002). We did not include sex into our models, because initial data exploitation showed no gender effect. We visualized the percentage of locations within a habitat feature compared to the corresponding CRW by using as a mosaic plot (Hofmann, Siebes, & Wilhelm, 2000) and predicted and plotted the results (package “raster”; Hijmans et al. (2017)).

#### 2.4.2 Landscape distance model

To test whether landscape features are tolerated by keeping certain distances, we used the same response variable as described above to build GLMMs using template model builder (package “glmmTMB”; Magnusson et al. (2016)) but used the distances to all seven landscape features in additive and interactive combination to the predictor variables “sex” and “time.” We also included ID as a random factor. All candidate models are listed in Table 3 in Appendix 2, and we compared and ranked the candidate models using AIC_c_ and model weights as described above. Prior to fitting the final model, we tested whether the predictor variables “distance to habitats features” were strongly correlated (Pearson's |*r*|> 0.7) using the Pearson's product–moment correlation coefficient panel for pairs function. No strong correlation was found between variables (Figure 6 in Appendix 1). Further, we explored the shape of the response variable for each distance variable to check for linearity (Austin, 2002) (Figure 7 in Appendix 1).

We built generalized additive models (GAMs) (Austin, 2002; Hastie & Tibshirani, 1987) using the binomial response variable as a function of the dependent distances to landscape features, each fitted with a smoothing spline with three degrees of freedom (package “mgcv”). When necessary, we transformed the predictor variables in the GLMMs with template model builder to model a second‐order polynomic relationship guided by visual inspection of the smoothing terms of the GAMs (Figure 7 in Appendix 1) (Klar et al., 2008). Second‐order polynomic relationships were found in all distance form landscape feature variables except for waterbodies and housing. Predictor variables were standardized by centering and z‐transforming the values. We predicted and plotted the results of the best model with the lowest AIC_c_ for each distance to landscape feature variable using the “predict” function of the “raster” package (Hijmans et al., 2017). All distance variables were cut off at the turning point of the fitted smoothing spline (test for linearity (Figure 7 in Appendix 1)) or at a maximum of 500 m as we assumed that further distances would not be biologically representative, based on the wallabies’ calculated 95% kernel home range (Table 2 in Appendix 2).

**Figure A3:**
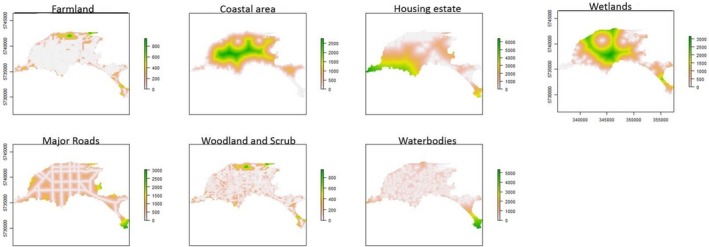
Distance maps of all landscape features used to build a stack for the prediction map of the distance to feature model

For both, the landscape use and distance model, we assessed model fit by calculating marginal and conditional R^2^ as outlined by Nakagawa and Schielzeth (2013). The marginal *R* ^2 ^(*R* ^2^ *m*) values refer to the variance explained by the fixed factors, and conditional *R* ^2^ (*R* ^2^ *c*) is the variance explained by both fixed and random factors. We used the best AIC_c_ model for each approach, the landscape use, and distance model, to extrapolate the results. Probability of wallaby occurrence on Phillip Island was extrapolated using the “predict” function, and we used a raster stack including the habitat map with all landscape features (landscape use model) and one raster layer for each landscape feature where each cell value indicates the distance to the represented landscape features (landscape distance model, Figure A3). The predicted maps were visualized using the “raster” and “pals” package (20 × 20 m resolution) (Hijmans et al., 2017; Wright, 2016).

## 3 RESULTS

**Figure 2:**
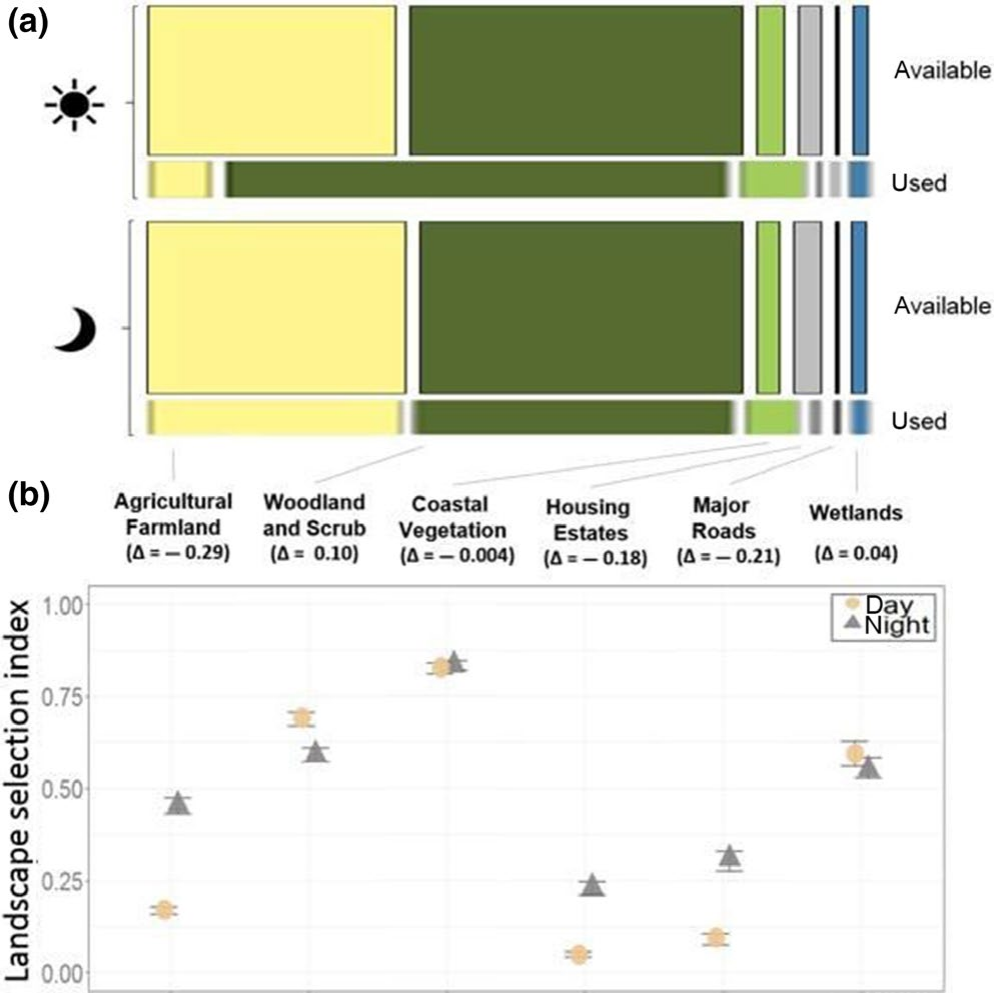
Wallaby landscape use versus availability on Phillip Island between 2015 and 2017. (a) Percentage (bar width) indicate usage (observed wallaby location) versus availability (simulated locations) located within six landscape features and compared between day and night. The height of the bars indicates the total number of locations within one of the four groups (ratio 1:5 (observed:simulated). (b) Predicted habitat selection index of wallabies during day and night. Greater values indicate that it was more likely for observed wallabies to use the habitat based on the availability of the habitat, and smaller values indicate that it was less likely for wallabies to use the respective habitat. Δ indicates the differences in the habitat use index between day–night. Error bars indicate 95% confidence intervals

In total, 22,947 GPS locations were recorded of which 10,649 were classified as day locations and 12,298 as night locations. During the day, most wallaby locations occurred in woodland and scrub (80.8%), while 7.7% were found in agricultural farmland. During the night, 36.7% of locations were found in farmland, and only 53.3% of locations in woodland and scrub. Further, wallaby presence increased from 0.4% locations during the day to 1.5% at night within housing estates and from 5.9% at night to 7.5% during the day in coastal vegetation. Wallabies occurred almost equally often on roads and in wetlands regardless of the time of the day (Figure 2).

**Figure 3:**
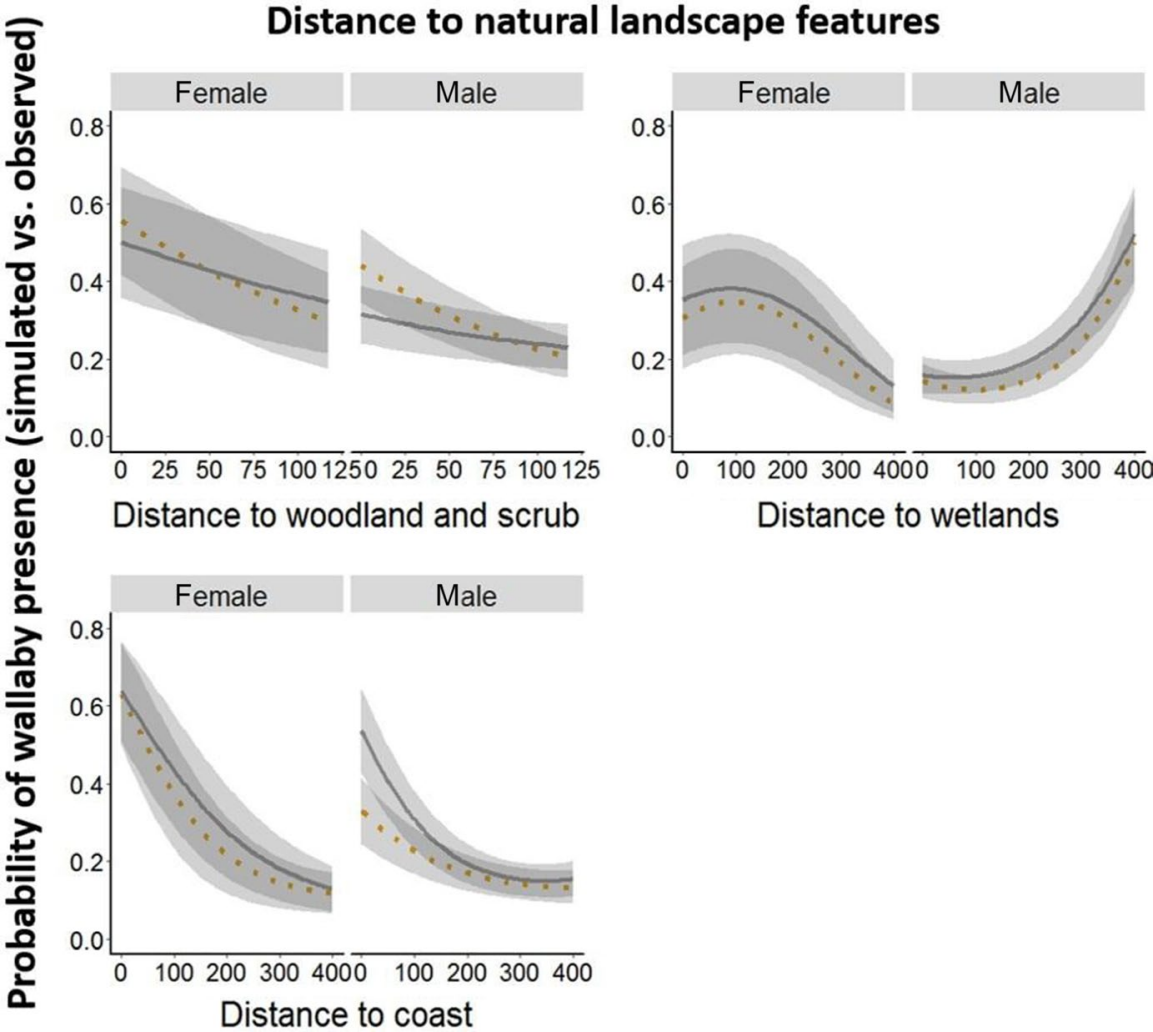
Prediction plots show probability of wallaby presence on Phillip Island between 2015 and 2017 in relation to distance to natural landscape features. Greater values indicate that is was more likely for wallabies to be within or near‐landscape features based on the availability of features, and smaller values indicate that it was less likely. The cutoff is 0.17 because we used five times more simulated walks than observed trajectories. Temporal variations (time) are shown in day (orange ‐ dashed line) and night (gray–solid line) in interaction with differences in sex (male, female)

**Figure 4:**
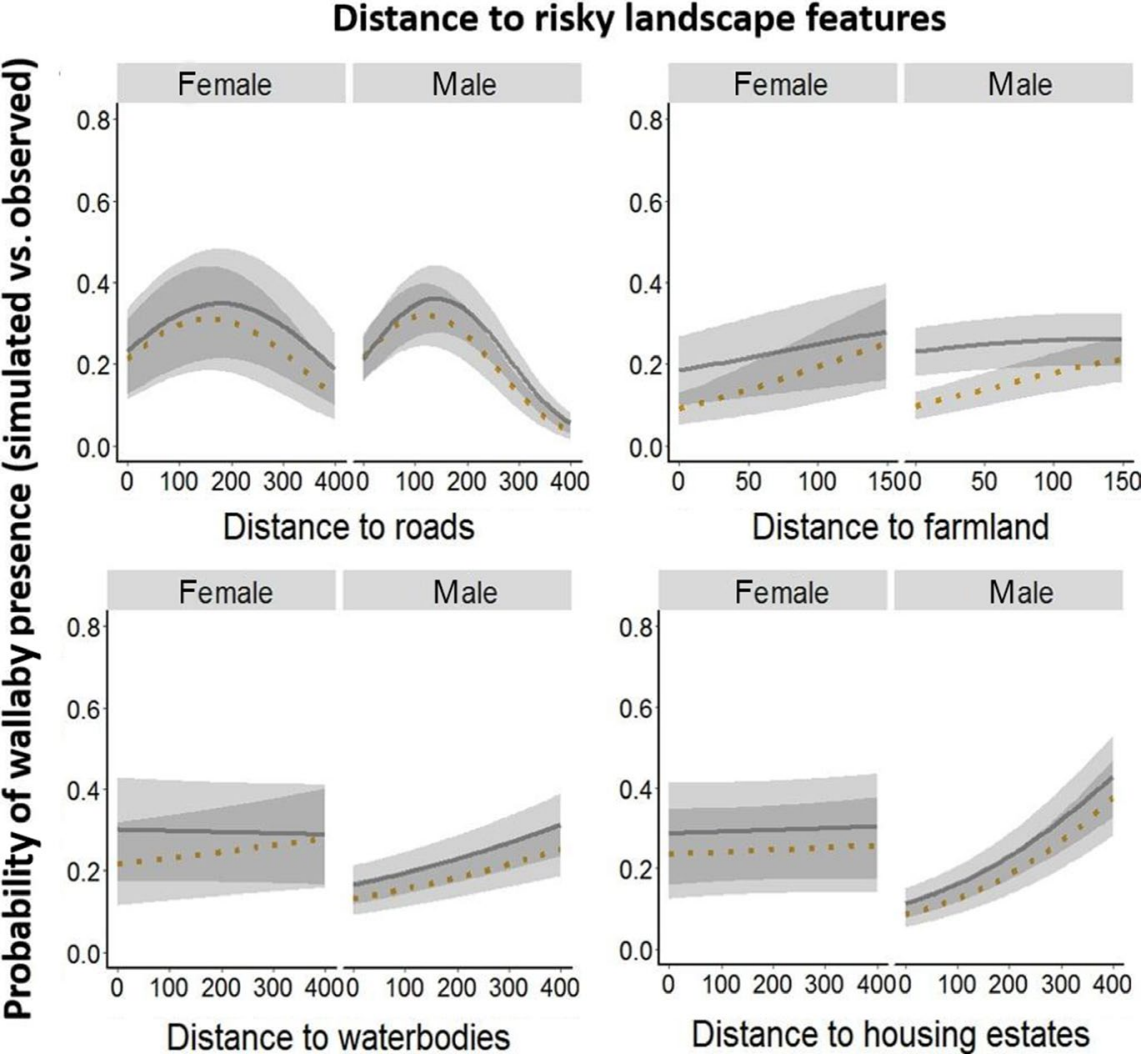
Prediction plots show probability of wallaby presence on Phillip Island between 2015 and 2017 in relation to distance to risky landscape features. Greater values indicate that is was more likely for wallabies to be within or near‐landscape features based on the availability of features, and smaller values indicate that it was less likely. The cutoff is 0.17 because we used five times more simulated walks than observed trajectories. Temporal variations (time) are shown in day (orange ‐ dashed line) and night (gray–solid line) in interaction with differences in sex (male, female)

**Figure 5:**
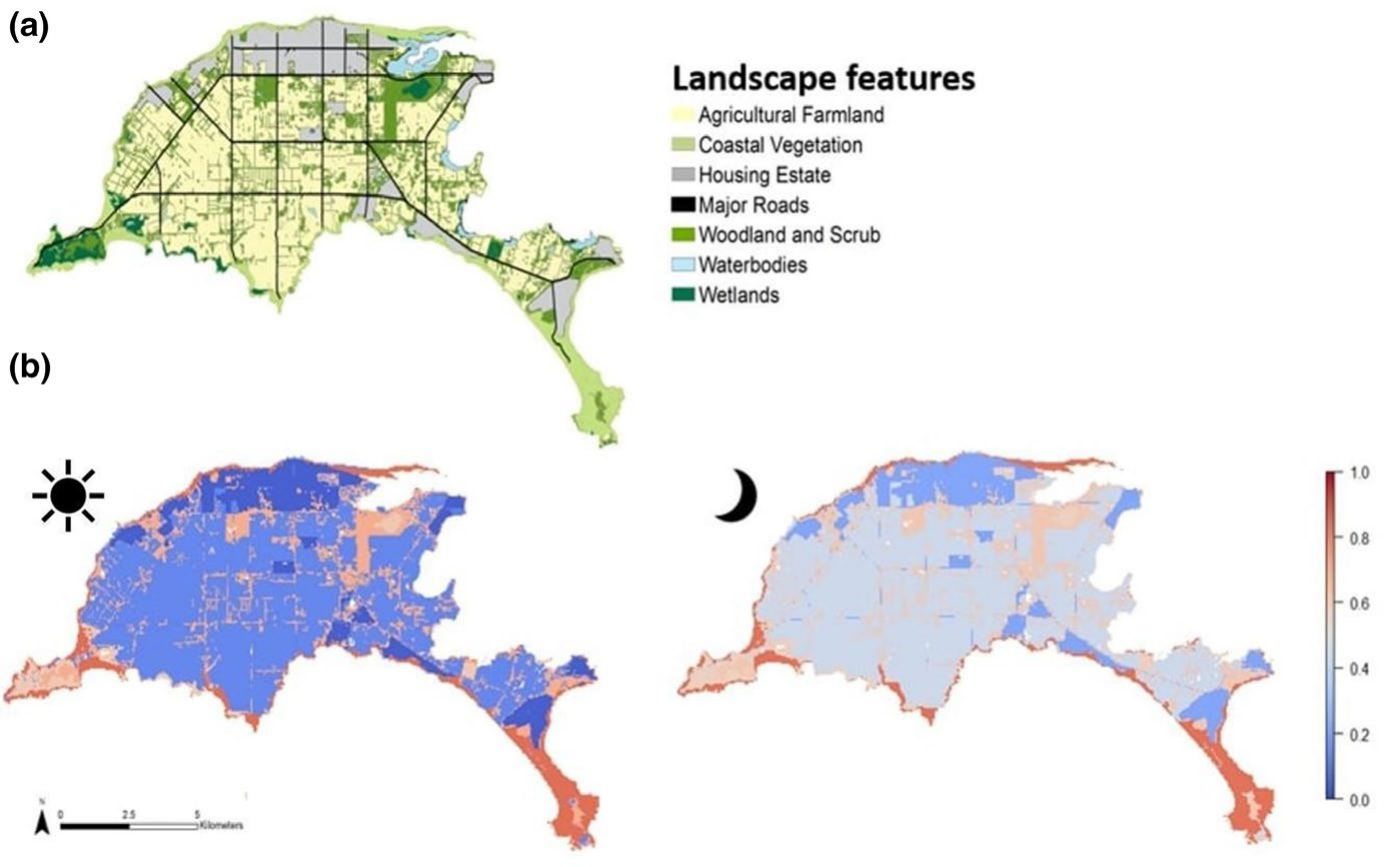
(a) Study site Phillip Island categorized using seven landscape features and corresponding (b) habitat suitability maps based on wallaby landscape feature selection use during the day (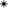) and at night (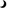). Probability values range from 0 to 1 with 0 being least suitable and 1 being most suitable habitat

Habitat use associated with wallaby trajectories differed from that of CRWs, indicating that wallabies selected specific habitats within their home range. The model consisting of all habitat feature variables in interaction with time was the most parsimonious landscape use model (Table 4 in Appendix 2). For the landscape distance model, the full model including all habitat variables in interaction with time and sex (three‐way interaction) was the best model in the model set with a model weight of 1 (Table 1, Table 1 in Appendix 2). Both models showed that the likelihood of finding wallabies in natural habitats (woodland and scrub, wetlands, and coastal vegetation) was high, especially during the day, whereas at night, the use of woodland and scrub decreased (Figures 2, 3, 4). Landscape features that may be perceived as risky by wallabies such as major roads, housing estates, and farmland were avoided, but the likelihood of finding wallabies near or within these features increased during the night (Figures 2, 3, 4). For example, in the landscape use model day and night differences were most noticeable regarding the use of farmland and woodland and scrub. During the night, wallabies were found in farmland and returned to woodland and scrub during the day. Both females and males selected areas further away from housings and waterbodies, especially during the day (0–400 m) (Figure 4). The prediction maps created from the best landscape use model predicted differences in the selection of landscape features during the day compared to night (Figure 5). During day and night, respectively, the maps predicted that it was most likely to find wallabies in coastal vegetation. Although roads and housing estates are avoided, the prediction maps revealed that wallabies can occur near housings and roads when using linear strips of bushland and scrub around roads (Figure 5).

**Table 1:** Model selection table identifying the most parsimonious model of resource selection by swamp wallabies on Phillip Island, southeastern Australia. Response of use (1 = observed wallaby location) and available (0 = simulated locations) to [a] the corresponding landscape feature variables (agricultural farmland, woodland and scrub, coastal vegetation, housing estate, major roads, wetlands) at each locations and time (day and night) differences and [b] distance to all landscape feature variables (agricultural farmland, woodland and scrub, coastal vegetation, housing estate, major roads, wetlands, waterbodies) in additive or interactive interaction to time and sex using [a] generalized linear mixed models (GLMM) and [b] GLMMs with template model builder. We included ID as a random factor for both model approaches. To rank models, we show Akaike's information criteria adjusted with corrections for finite sample size (AIC_c_) and the null model. For all other models in the model set, the difference in AIC_c_ units to the best model (ΔAIC_c_) was >20. Model weights (*w*) provide conditional probabilities for each model. The marginal and conditional *R* ^2^ is given as a measurement of fit. A detailed list of all models is shown in Table 3 in Appendix 2, and model estimates and standard errors are shown in Table 4 in Appendix 2 and Table 4 in Appendix 2

Variable	Model sets	*df*	ΔAIC_c_	*w*	logLik	*R* ^2^ *m*(*R* ^2^ *c*)
[a] Landscape use model						
Response	Landscape feature variables^a^ × time	13	0	1	−156008	0.23 (0.26)
	NULL	2	35,278.5	0	−173658	
[b] Landscape distance model						
Response	(Distance to landscape feature variables) × time × sex	53	0	1	−147375	0.26 (0.85)
	NULL	2	52,988.4	0	−173920	

a Because the landscape feature “waterbodies” was classified as unsuitable habitat for wallabies, only six habitat variables are used in the landscape use model.

## 4 DISCUSSION

In this study, we investigated whether wallabies living in a human‐modified landscape select different landscape features during the day and night, based on the level of risks that these landscape features may pose to wallabies. Our results show that wallabies were more likely to select natural landscape features with high cover such as woodland and scrub, wetlands, and coastal vegetation which supports our prediction. Our findings are in line with other studies where cover is an important resource for swamp wallabies (Coulson, Alviano, Ramp, & Way, 1999; Di Stefano et al., 2009; Troy et al., 1992; Wood, 2002) providing shelter, resting sites, and food. A study conducted by Lunney and O'Connell (1988) for example took place in logged and unlogged forest, where the selection of landscape types was examined for three macropods, including swamp wallabies, showing that areas with higher shrub cover and gullies were selected. Further we provide evidence that linear vegetation strips represented as bushland and scrub are suitable, especially during the day. It is likely that vegetation strips are used by wallabies as refuge areas and to move between remnant vegetation patches which is common in many species (Haddad et al., 2003; LaPoint, Gallery, Wikelski, & Kays, 2013) including macropods (Arnold, Steven, Weeldenburg, & Smith, 1993). We also anticipate that these strips provide cover to reduce perceived risks when moving within or near unsuitable habitat such as roads and farmland.

Although we would have expected woodland and scrub to be the most suitable landscape features for wallabies based on existing literature (Edwards & Ealey, 1975; Lunney & O'Connell, 1988; Troy et al., 1992; Wood, 2002), our maps predict that the suitability for coastal vegetation is higher. However, the studies listed above have been conducted in forest with high vegetation cover. In our study area, coastal vegetation is available in addition to woodlands which might explain different preferences of wallaby habitat selection. Most coastal areas are far away from roads and housing estates, provide sufficient cover and food resources, and may therefore be more suitable than other landscape features. On Phillip Island, linear vegetation strips and smaller patches of woodlands and scrub are often surrounded by habitats of high disturbance levels such as roads and farmland and larger areas of remnant and revegetated woodlands are popular for outdoor recreational activities and are sometimes located adjacent to housing. Hence, within these areas, wallabies may perceive risks, such as human disturbance on a regular basis and may therefore select coastal vegetation over woodlands and scrub. Woodlands and scrub mainly represent small patches, functioning as refuge areas but are surrounded by landscape features that pose higher risks. Coastal vegetation, however, represents similar vegetation but provide more continuously structures with low risks.

Further, we showed that wallabies avoided risky areas including major roads, farmland, housing estates, and waterbodies. These findings are consistent with studies on cervids, which are ecological analogues to macropods (Jarman, 1991) as cervids similarly avoid anthropogenically disturbed areas (Coulon et al., 2008; Webb, Dzialak, Harju, Hayden‐Wing, & Winstead, 2011). We also showed that the perception of risk can vary between day and night and the findings of both models support our predictions. Similar observations have been recorded in other studies investigating macropods (Johnson, 1980; Stirrat, 2003; Swan et al., 2008) and cervids (Bjørneraas et al., 2011; Godvik et al., 2009; Lykkja et al., 2009). The study of Bonnot et al. (2012), for example, showed that roe deer spent more time in woodlands for cover during the hunting season and avoided high‐crops, an important source of cover and food, during the day. Further, Di Stefano et al. (2009) showed that swamp wallabies alter their selection of habitat within a 24‐hr cycle within recently disturbed landscapes. Swamp wallabies chose areas with more lateral cover during the day compared to night when comparing timber forests of varying harvesting ages (Di Stefano et al., 2009). We showed that although it was unlikely to find wallabies near risky landscape features (major roads, housings, farmland, waterbodies, and agricultural farmland), the likelihood was higher during the night. Hence, our finding suggests that wallabies have a different behavior based on the perception of risk, which is lower at night when we assume that human activity is reduced which is well supported by other studies. For example, the choice of habitat in roe deer is influenced by the proximity of human‐disturbed areas such as roads and housings (Bonnot et al., 2012). Areas of high risk such as open grassland were used more during the day when areas of high disturbances (roads and dwellings) were further away (Bonnot et al., 2012). Further, results of M. Fischer, M. Stillfried, G. Coulson, D. R. Sutherland, S. Kramer–Schadt, and J. Di Stefano (in prep) indicated that it was less likely for the same wallabies to cross roads, a risky venture, than it was by chance. Roads in particular pose the risk of mortality caused by animal‐vehicle collisions and may be recognized and avoided (Rondinini & Doncaster, 2002; Shepard, Kuhns, Dreslik, & Phillips, 2008). However, there is evidence that swamp wallabies are attracted to roadside vegetation because of the vegetations’ availability or high nutritional value (Osawa, 1990).

There is evidence that swamp wallabies are opportunists, taking advantage of novel food resources when they become available (Osawa, 1990). A high variety of food resources, for instance, is found in areas of high vegetation cover. If foraging behavior is highly correlated with habitat selection, it is unclear whether wallabies move into and select different landscape features to access alternative food resources when their main source is depleted or unavailable in a changing environment. Here, we recorded a differing circadian pattern, showing that the selection of woodland and scrub decreased during the night when wallabies were found nearer or within farmland. Because woodland and scrub provide shelter and food, this result suggests that wallabies move further away from these habitats during the night to feed on other, perhaps richer resources such as pasture but return to sheltered areas during the day for feeding and resting which was also observed by Edwards and Ealey (1975). In addition, Johnson (1975) showed that macropods sometimes occur in high abundances near cover, but rarely move more than 70 m onto pasture from a forest edge and similar behavior was observed for the wallabies within our study site. Edge‐oriented behaviors are often attributed to the increased richness of resources found on vegetation edges (Odum, Odum, & Andrews, 1971), however within our study site, boundaries between landscape features are sharp and we conclude that wallabies were attracted by the different resources (shelter vs. food) provided within each landscape feature, but avoided pasture during the day due to higher risk. Our findings suggest that the high selection of cover during the day and an increase in selection of farmland during the night are likely due to swamp wallabies optimizing the trade‐off between the selection of another rich food source, such as pasture (Johnson, 1980) and risk and stress avoidance. Such temporal transition is shown by many macropods (Le Mar & McArthur, 2005; Swan et al., 2008) and is often explained as behavioral responses to potential predators. Because the abundance of natural predators for wallabies is low within our study site, it is likely that in this human‐dominated landscape, risk is caused by human presence.

The landscape distance model also revealed differences in habitat selection between sexes, therefore supports our second prediction. The likelihood of finding wallabies near waterbodies was low, especially for males and during the day. Similar results were found for wetlands and housing which often also contain artificial water sources and it may be safer to gain access to these resources during the night. Changes in habitat selection of reproducing females have been found in moose (Bjørneraas et al., 2011) where open and food rich areas were avoided by females, when the calve was under one month old. Lactating marsupial females have been shown to have a higher water turnover than nonlactating females (Kennedy & Heinsohn, 1974). Here, we suggest female wallabies may need access to water more regularly when they have young (Table 2 in Appendix 2) and are therefore less likely to show temporal variations in their circadian resource selection pattern. However, further studies are necessary to clarify behavioral differences in males and females in response to distance to natural and risky water sources.

The prediction maps identify areas where humans are most likely to encounter swamp wallabies, such as roadside vegetation and woodland and scrub adjacent to housing estates. We also predicted and visualized how this can vary on a circadian basis, which can enhance management planning. Our findings are also supported by a study undertaken within the same study site, estimating the density of swamp wallabies on Phillip Island by observation using distance sampling along line transects (D. R. Sutherland, in prep). The study indicates that density is highest in some coastal areas, remnant bushland patches and vegetation strips, and lowest in farmland and housing estates.

## 5 CONCLUSION

We present how wallabies living in a human‐modified landscape select landscape feature on a circadian basis to optimize the use of resources. This study enhances our ecological understanding of swamp wallabies living on Phillip Island, which represents a human‐modified landscape. We show that wallabies choose habitats with high cover and avoid areas that may expose wallabies to greater risk such as roads, housing estates, waterbodies, and agricultural farmland. These findings can be used to enhance management actions such as fencing along paddocks to mitigate pasture loss. Further, we demonstrate that the use of these landscape features varies in a circadian pattern. Landscape features likely to be perceived as risky during the day are selected more during the night when we assume that risks, such as human disturbances are lower. More generally, our results show that habitat selection in a human‐modified landscape can vary within a circadian cycle to optimize the trade‐off between accessing high‐quality resources and reduced risks. In some cases, management of species in human‐modified landscapes may benefit if circadian differences in behavior are taken into account.

## CONFLICT OF INTEREST

The authors declare that there is no conflict of interest.

## AUTHORS CONTRIBUTION

MF, JDS, GC, and DS conceptualized the data. MF performed data curation. MF, MS, SKS, PG involved in formal analysis. DS involved in funding acquisition. MF administered the project. JDS, DS, and GC supervised the study. MF visualized the data. MF involved in writing—original draft. MF, MS, SKS, PG, JDS, and DS involved in writing—review and editing.

## Figures and Tables

**Figure A1:**
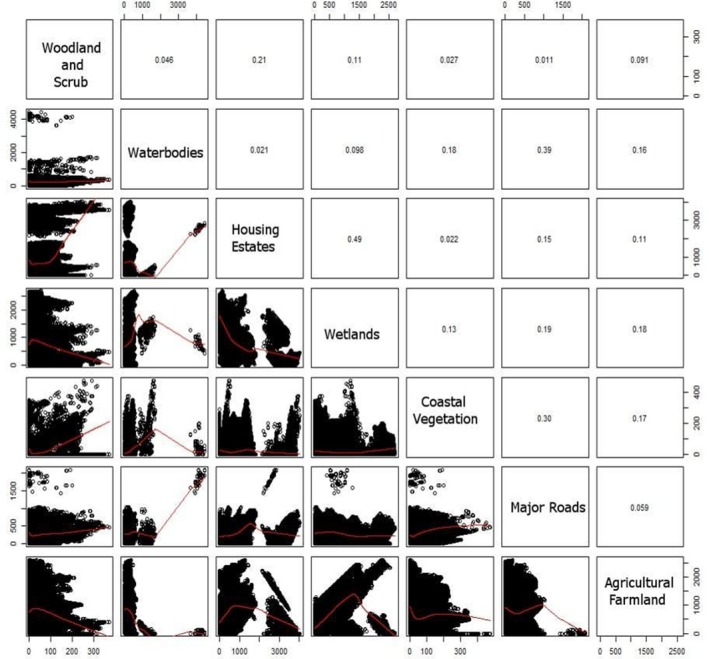
Correlation plot to check for correlation between distances to different landscape features. We used Pearson correlation coefficients to measure correlation between variables. Correlating variables with |*r*| > 0.7 would have been excluded from the final model but none of our predictor variables showed correlations

**Figure A2:**
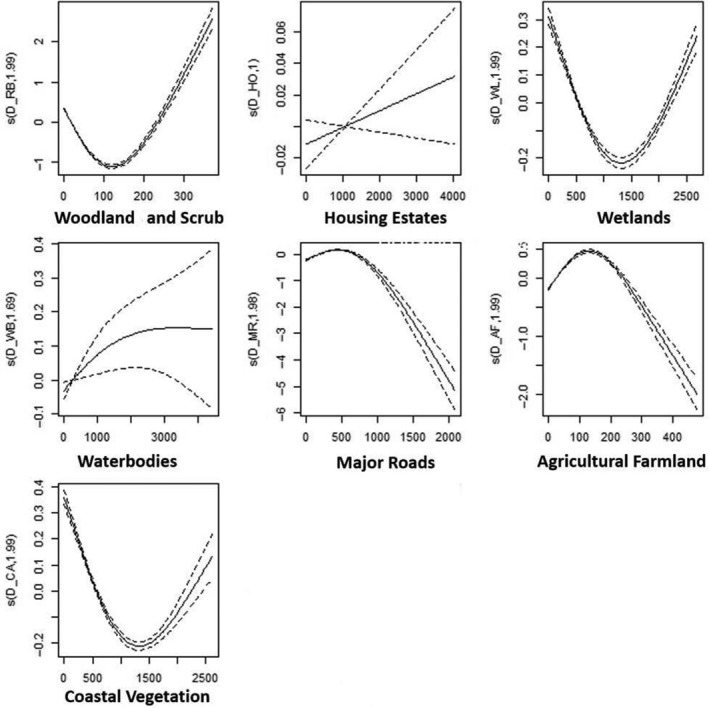
Generalized additive model (GAM) plots of the nonlinear estimator for distance to habitat feature variables to visualize linearity assumption. We used observed wallaby locations (1) versus simulated locations (0) as a binary response variable and fitted smoothing splines with 3 degrees of freedom to model the effect on all habitat variables (woodland and scrub, housing estates, wetlands, waterbodies, major roads, agricultural farmland, coastal vegetation). If variables were nonlinear, we included the quadratic term of the variables in the final models, which was the case for all variables but waterbodies and housing estates

## Data Availability

The datasets generated during and/or analyzed during the current study are available at the Movebank Data Repository (https://www.datarepository.movebank.org) as "Fischer et al. (2019) Swamp wallabies on Phillips Island, Australia". https://doi.org/10.5441/001/1.6ss053tn. Access to the data has been embargoed for one year from the date of publication.

## APPENDIX 2

**Table A1:** Overview of all wallabies sampled including sex, pouch young, weight, tracking duration, number of GPS locations, and home range area (95% kernel estimate)

ID	Sex	Pouch young	Weight (kg)	Tracking duration	Home range (ha)	GPS points	
						Day	Night
MF001	♂	NA	14.8	21.03.2015–30.03.2015	28.7	312	379
MF006	♂	NA	14.8	12.01.2015–17.01.2015	10.7	52	40
MF010	♂	NA	15.8	21.03.2015–27.03.2015	13.6	215	314
MF011	♂	NA	15.1	22.03.2015–30.06.2015	16.5	168	274
MF014	♂	NA	10.8	23.05.2015–02.04.2015	4.8	290	505
MF015	♂	NA	10.3	29.06.2015–07.07.2016	5.5	220	444
MF017	♂	NA	14.2	28.06.2015–08.06.2015	31.7	295	519
MF019	♂	NA	14.7	17.07.2015–24.07.2015	9.2	168	299
MF023	♂	NA	13.9	06.01.2016–15.01.2016	22.8	252	182
MF030	♂	NA	16.2	16.01.2016–21.01.2016	23.9	165	175
MF031	♂	NA	13.1	28.02.2016–10.03.2016	11.1	396	431
MF039	♂	NA	15.9	28.04.2016–08.05.2016	18.6	306	463
MF044	♂	NA	16.9	13.02.2017–24.02.2017	11.1	457	383
MF047	♂	NA	13.9	12.02.2017–20.02.2017	31.2	236	309
MF049	♂	NA	19.3	04.03.2016–14.03.2016	15.7	222	308
MF050	♂	NA	18.4	15.01.2017–20.01.2017	NA	4	9
MF051	♂	NA	10.6	16.01.2017–24.01.2017	15.1	111	105
MF052	♂	NA	18.3	12.02.2017–21.02.2017	44.8	321	311
MF053	♂	NA	16.0	12.02.2017–22.02.2017	14.5	461	362
MF048	♂	NA	11.0	21.02.2016–28.02.2016	18.2	65	66
MF061	♂	NA	9.7	14.02.2017–20.02.2017	12.7	93	93
MF055	♂	NA	17.9	13.02.2017–23.02.2017	7.2	464	424
MF064	♂	NA	10.1	02.03.2017–09.03.2017	12.6	222	228
MF066	♂	NA	12.0	14.02.2017–23.02.2017	23.8	41	46
MF068	♂	NA	11.8	04.03.2017–10.03.2017	4.1	226	224
MF069	♂	NA	14.4	16.03.2017–25.03.2017	11.4	286	303
MF070	♂	NA	14.4	16.03.2017–24.03.2017	14.8	296	327
MF002	♀	Yes	10.1	16.01.2015–20.01.2015	11.7	122	69
MF009	♀	No	8.9	01.05.2015–18.05.2015	9.7	194	237
MF013	♀	Yes	13.0	22.03.2015–02.04.2015	7.2	282	470
MF016	♀	Yes	13.8	27.06.2015–07.07.2015	12.6	270	483
MF018	♀	Yes	12.6	17.07.2015–25.07.2015	11.3	141	147
MF021	♀	No	11.7	15.12.2015–24.12.2015	5.1	427	307
MF022	♀	No	11.1	17.12.2015–23.12.2015	12.3	85	56
MF024	♀	Yes	10.5	17.01.2016–20.01.2016	23.6	7	18
MF028	♀	Yes	12.8	08.02.2016–16.02.2016	13.6	299	332
MF029	♀	Yes	13.2	08.02.2016–21.02.2016	16.7	337	346
MF032	♀	No	12.8	30.03.2016–09.03.2016	15.4	299	402
MF033	♀	Yes	10.3	31.03.2016–03.04.2016	15.3	7	59
MF038	♀	Yes	11.0	28.04.2016–03.05.2016	4.0	134	203
MF040	♀	Yes	11.3	28.04.2016–04.05.2016	90.7	176	253
MF043	♀	Yes	9.5	14.02.2017–16.02.2017	18.1	1	19
MF046	♀	Yes	10.2	04.03.2017–10.03.2017	4.0	66	130
MF054	♀	No	14.6	13.02.2017–22.02.2017	22.4	421	376
MF056	♀	Yes	13.5	15.02.2017–23.02.2017	13.9	280	264
MF057	♀	No	9.2	13.02.2017–23.02.2017	5.2	449	381
MF058	♀	No	12.4	14.02.2017–17.02.2017	0.1	54	20
MF063	♀	No	11.3	15.02.2017–21.02.2017	9.9	254	204

**Table A2:** List of model sets using a [a] binomial generalized linear mixed model (GLMM) and [b] GLMMs with template model builder. We used observed wallaby data (1) versus simulated trajectories (0) as a binary response variable. [a] Landscape use model: For each location, we used landscape variables (seven levels) and time (day vs. night) as predictor variables. Because the landscape feature “waterbodies” was classified as unsuitable habitat for wallabies, only six landscape feature variables are used in the model. We included ID as a random factor. [b] Distance model: We used the same response variable and random factor as in the landscape use model. As predictor variables, we used distances to each landscape variable and sex and time. We included nonlinear variables as a quadratic term, which was the case for all variables except for waterbodies and housing (Figure 7 in Appendix 1). We applied the scale function (R package “base”) to standardize all landscape features

Model	Predictor variables	Random factor
[a] Landscape use model		
Null		ID
Model 1	Woodlands + Coastal + Housing + Wetlands+Roads + Farmland	ID
Model 2	Woodlands + Coastal + Housing + Wetlands + Roads + Farmland + time	ID
Model 3	Woodlands + Coastal + Housing + Wetlands + Roads + Farmland × time	ID
[b] Distance model		
Null		ID
Model 1	(Woodlands + Coastal + Housing + Wetlands + Waterbodies + Roads + Farmland + Woodlands^2^ + Coastal^2^ + Wetlands^2^ + Roads^2^ + Farmland^2^)	ID
Model 2	(Woodlands + Coastal + Housing + Wetlands + Waterbodies + Roads + Farmland + Woodlands^2^ + Coastal^2^ + Wetlands^2^ + Roads^2^ + Farmland^2^) × time	ID
Model 3	(Woodlands + Coastal + Housing + Wetlands + Waterbodies + Roads + Farmland + Woodlands^2^ + Coastal^2^ + Wetlands^2^ + Roads^2^ + Farmland^2^) + time	ID
Model 4	(Woodlands + Coastal + Housing + Wetlands + Waterbodies + Roads + Farmland + Woodlands^2^ + Coastal^2^ + Wetlands^2^ + Roads^2^ + Farmland^2^) + time + sex	ID
Model 5	(Woodlands + Coastal + Housing + Wetlands + Waterbodies + Roads + Farmland + Woodlands^2^ + Coastal^2^ + Wetlands^2^ + Roads^2^ + Farmland^2^) + time × sex	ID
Model 6	(Woodlands + Coastal + Housing + Wetlands + Waterbodies + Roads + Farmland + Woodlands^2^ + Coastal^2^ + Wetlands^2^ + Roads^2^ + Farmland^2^) × time + sex	ID
Model 7	(Woodlands + Coastal + Housing + Wetlands + Waterbodies + Roads + Farmland + Woodlands^2^ + Coastal^2^ + Wetlands^2^ + Roads^2^ + Farmland^2^) × time × sex	ID

**Table A3:** Model estimates, standard errors (*SE*), and *p*‐values of the most parsimonious model for the generalized linear mixed models (GLMMs). We used observed wallaby data (1) versus simulated trajectories (0) as a binary response variable and the best model includes six landscape feature variables and “time” (day/night) as predictor variables. The model included animal ID as a random factor

Model	Variables	Estimate	*SE*	*p*‐Value
Woodlands + Coastal + Housing + Wetlands + Roads + Farmland × time	Woodlands	2.40	0.02	<0.01
	Coastal	3.17	0.03	<0.01
	Housing	−1.35	0.06	<0.01
	Roads	−0.65	0.10	<0.01
	Wetlands	1.99	0.05	<0.01
	night	1.41	0.02	<0.01
	Woodlands:night	−1.85	0.02	<0.01
	Coastal:night	−1.37	0.04	<0.01
	Housing:night	0.35	0.07	<0.01
	Roads:night	0.00	0.12	0.98
	Wetlands:night	−1.61	0.06	<0.01

**Table A4:** Model estimates, standard errors (*SE*), and *p*‐values of the most parsimonious model for the generalized linear mixed models with template model builder (glmmTMBs). We used observed wallaby data (1) versus simulated trajectories (0) as a binary response. The best model includes all distances to seven landscape features and “time” (day and night) and “sex” as predictor variables. The model included animal id as a random factor. We standardized all landscape variables by centering and *z*‐transforming the values

Model	Variable	Estimate	*SE*	*p*‐Value
(Woodlands + Coastal + Housing + Wetlands + Waterbodies + Roads + Farmland + Woodlands^2^ + Coastal^2^ + Wetlands^2^ + Roads^2^ + Farmland^2^) + time × sex	Woodlands	−2.23	0.03	<0.01
	Coastal	−2.24	0.08	<0.01
	Housing	−0.15	0.05	<0.01
	Wetlands	0.47	0.09	<0.01
	Waterbodies	0.08	0.03	0.02
	Roads	1.56	0.05	<0.01
	Farmland	2.17	0.04	<0.01
	Woodlands^2^	1.41	0.03	<0.01
	Coastel^2^	0.92	0.07	<0.01
	Wetlandss^2^	−1.71	0.11	<0.01
	Roads^2^	−2.25	0.07	<0.01
	Farmland^2^	−1.53	0.04	<0.01
	night	0.52	0.02	<0.01
	male	−1.24	1.04	0.23
	Woodlands:night	1.14	0.04	<0.01
	Coastal:night	0.55	0.08	<0.01
	Housing:night	−0.11	0.02	<0.01
	Wetlands:night	−0.66	0.10	<0.01
	Waterbodies:night	−0.14	0.03	<0.01
	Roads:night	0.13	0.05	<0.01
	Farmland:night	−1.47	0.05	<0.01
	Woodlands^2^:night	−0.93	0.03	<0.01
	Coastal^2^:night	−0.41	0.06	<0.01
	Wetlands^2^:night	0.70	0.10	<0.01
	Rroads^2^:night	0.12	0.06	0.07
	Farmland^2^:night	1.17	0.05	<0.01
	Woodlands:male	0.04	0.05	0.46
	Coastal:male	0.33	0.10	<0.01
	Housing:male	1.48	0.06	<0.01
	Wetlands:male	−0.62	0.11	<0.01
	Waterbodies:male	0.27	0.04	<0.01
	Roads:male	0.16	0.06	<0.01
	Farmland:male	−0.38	0.05	<0.01
	Woodlands^2^:male	0.26	0.05	<0.01
	Coastal^2^:male	0.22	0.10	0.03
	Wetlands^2^:male	2.90	0.14	<0.01
	Roads^2^:male	−0.75	0.09	<0.01
	Farmland^2^:male	−0.03	0.05	0.48
	Night:male	−0.06	0.02	0.01
	Woodlands:night:male	0.02	0.06	0.77
	Coastal:night:male	−1.31	0.11	<0.01
	Housing:night:male	0.06	0.03	0.02
	Wetlands:night:male	0.90	0.12	<0.01
	Waterbodies:night:male	0.17	0.04	<0.01
	Roads:night:male	0.27	0.07	<0.01
	Farmland:night:male	0.03	0.06	0.64
	Woodlands^2^:night:male	0.06	0.06	0.34
	Coastal^2^:night:male	0.94	0.10	<0.01
	Wetlands^2^:night:male	−1.00	0.12	<0.01
	Roads^2^:night:male	−0.27	0.10	<0.01
	Farmland^2^:night:male	−0.21	0.06	<0.01
